# Applying PyRosetta molecular energies to separate properly oriented protein models from mirror models, obtained from contact maps

**DOI:** 10.1007/s00894-016-2975-3

**Published:** 2016-04-23

**Authors:** Monika Kurczynska, Ewa Kania, Bogumil M. Konopka, Malgorzata Kotulska

**Affiliations:** Faculty of Fundamental Problems of Technology, Department of Biomedical Engineering, Wroclaw University of Science and Technology, Wybrzeze Wyspianskiego 27, 50-370 Wroclaw, Poland; Biotechnology Center, Dresden University of Technology, Tatzberg 47/49, 01307 Dresden, Germany

**Keywords:** Contact maps, Energy terms, Mirror images, Protein structure reconstruction, Protein structure

## Abstract

Reconstructing protein structure based on contact maps leads to two types of models: properly oriented models and mirror models. This is due to the fact that contact maps do not include information on protein chirality. Therefore, both types of model orientations share the same contact map and are geometrically allowed. In this work, we verified the hypothesis that some of the energy terms calculated by PyRosetta could be useful to distinguish between properly oriented and mirror models. We studied 440 models of all-alpha protein domains reconstructed manually from their contact maps, where 50 % of the models were properly oriented and 50 % had mirror orientation. We showed that dihedral angles and energy terms, based on the probability of specific geometrical arrangement of the residues, differed significantly for properly oriented and mirror models.

**Electronic supplementary material:**

The online version of this article (doi:10.1007/s00894-016-2975-3) contains supplementary material, which is available to authorized users.

## Introduction

Protein structure reconstruction based on contact maps is a well-known approach to protein structure prediction [1–3]. As methods for contact map prediction improve [4–8], structure prediction methods based on contact maps have also been further developed [9–12]. Methods based solely on contact maps encounter the problem of generating mirror image structures. This is due to the fact that the information encoded in contact maps does not include chirality, and the same interatomic distances can be satisfied by both properly oriented structures or their mirror images [13, 14]. A number of methods have been used to overcome this problem, which occurs during model generation, by adding a chirality-related term to the cost functions that guide model generation [1, 3, 15]. Another solution is adjustment of torsion angles in an additional processing step [2]. Vendruscolo et al. [3] reported that chirality terms usually enable properly oriented and mirror structures to be distinguished in cases of proteins rich in alpha-helices, but this does not hold for all-beta proteins. For the latter group of structures, a post-generation filtering procedure has been proposed, in which generated structures are clustered into two groups, and the group closer to the native structure, in terms of root mean square deviation (RMSD), is retained. A similar procedure was used by Duarte et al. [10], where all models were ranked relative to the native structure based on their RMSD, and one-third of the models with the lowest RMSD were taken as properly oriented. Such an approach may introduce a bias in the final results since it tends to select better models. This affects the efficiency of the modelling method when it is evaluated based on the resulting models. Moreover, in real life the native structures of target proteins are not available, therefore an alternative method for distinguishing between properly oriented and mirror structures is needed.

Although contact-based reconstruction methods in general treat mirror structures as computational artefacts, recent studies with molecular dynamics methods showed that mirror structures may be stable and thermodynamically competitive conformations of a protein [16, 17].

Our ultimate goal is to propose a method for filtering out mirror structures resulting from protein reconstruction based on contact maps. In this work, we investigate the differences between properly oriented and mirror structures and verify whether it is possible to distinguish these models based on energy terms, and select the most suitable terms for their separation.

## Materials and methods

### Data set

First, protein contact maps for 55 domains, which were randomly chosen from the SCOP [18] all-alpha superfamily, were derived with PconPy [19]. Only all-alpha domains were chosen since their mirror images and properly oriented structures are easily distinguishable by visual inspection, through the handedness of their helices. Next, the contact maps were used as an input for C2S_pipeline [11] to reconstruct structural models. For each selected domain, 50 models were generated, and their RMSD distributions investigated. Domains for which the histograms showed two non-overlapping distributions were selected for the next processing step, from which 24 domains were selected. Finally, 11 domains for which at least 20 properly oriented models and 20 mirror models were obtained were chosen for further analysis.

The orientations of models (properly oriented or mirror) were assessed by visual inspection using PyMol [20]. In this step, each of 1200 models was assessed manually to ensure proper assignment of orientation. The Ramachandran plots of the models were prepared with Rampage [21]. The number of positive dihedral angles, *Φ*, of the models was calculated using Biopython [22, 23]. The ratio of the number of positive dihedral angles *Φ*, to all dihedral angles *Φ* is denoted as the *Φ*^+^ratio.

### Energy/scoring function

We used the *talaris2013* energy score function from the PyRosetta package [24]. The total energy of a model is the weighted sum of 16 energy terms. The energy terms, along with their short descriptions, are listed in Table 1. The energy term *dslf_fa13* was not included in the analyses because, in the domains included in the study, no disulfide bonds were present.

**Table 1:** Description of the energy terms

Energy term shortcut	Energy term description
*fa_atr*	Lennard-Jones attractive
*fa_rep*	Lennard-Jones repulsive
*fa_sol*	Lazardis-Karplus solvation energy
*fa_intra_rep*	Lennard-Jones repulsive between atoms in the same residue
*hack_elec*	Coulomb interaction
*pro_close*	Proline ring closure energy
*hbond_sr_bb*	Backbone-backbone hydrogen bonds close in primary sequence
*hbond_lr_bb*	Backbone-backbone hydrogen bonds distant in primary sequence
*hbond_bb_sc*	Sidechain-backbone hydrogen bond energy
*hbond_sc*	Sidechain-sidechain hydrogen bond energy
*dslf_fa13*	Disulfide bonds energy
*rama*	Ramachandran preferences
*omega*	Omega dihedral in the backbone
*fa_dun*	Internal energy of sidechain rotamers as derived from Dunbrack’s statistics
*p_aa_p*	Probability of amino acid at Φ and Ψ
*ref*	Reference energy for each amino acid
*total*	Final score (total energy)

### Assessment of energy terms usability

**Fig. 1:**
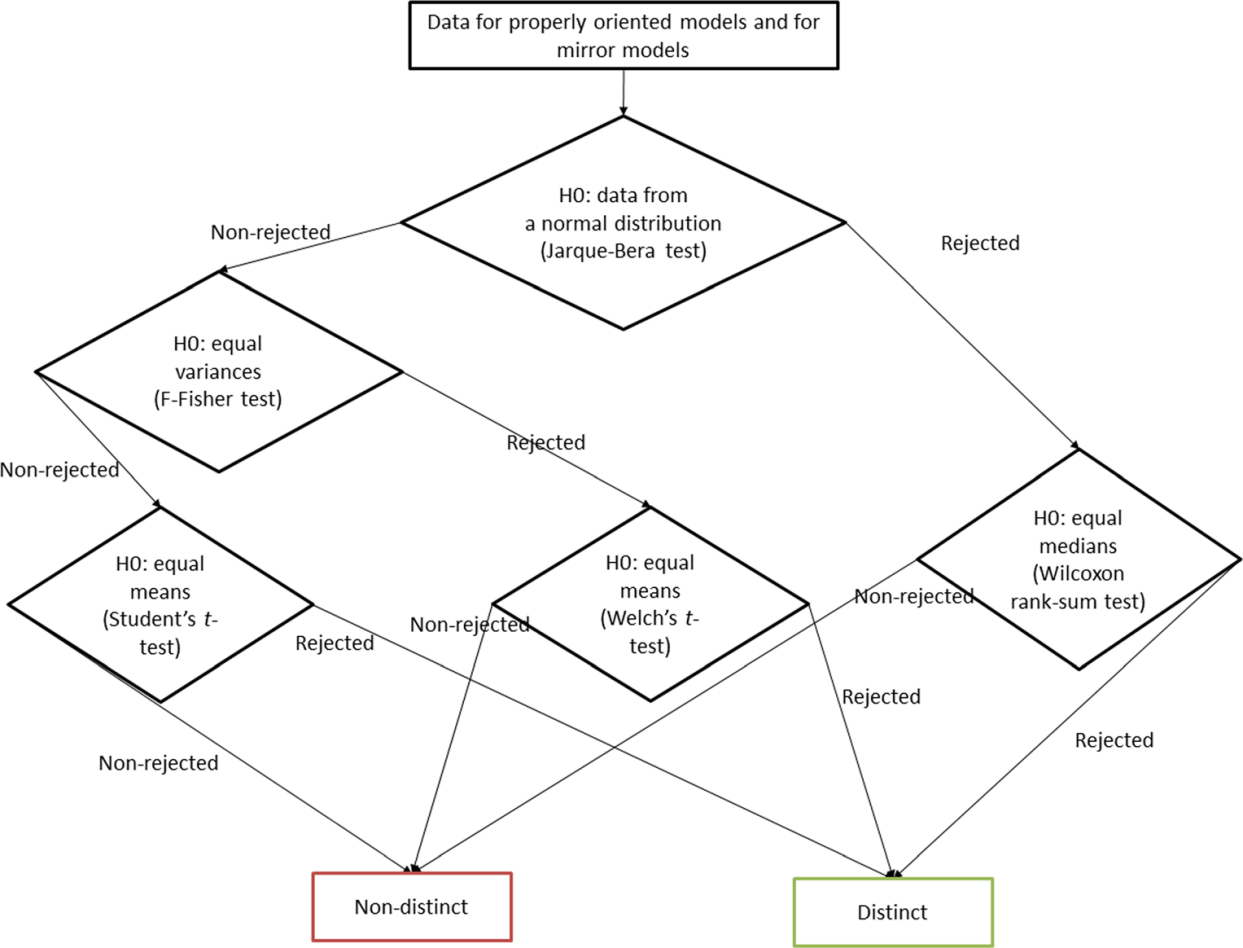
Schema of statistical analyses of the energy terms in the groups of properly oriented models and the mirror models

The basic hypothesis to be verified was whether a certain energy term is significantly different in properly oriented and mirror models of a protein domain. The statistical analyses were performed with MATLAB. The schema of the energy terms comparison between the properly oriented and mirror models is shown in Fig. 1. To visualize the range of the energy term values for the models, the following formula was used:

$$ NM{T}_i=\frac{{\overline{T}}_i}{MAX\left({\overline{T}}_1,{\overline{T}}_{i+1},\dots, {\overline{T}}_{i+n}\right)} $$

where: *NMT*_I_ is the normalized energy term mean value for the *i*^th^ domain, ${\overline{T}}_i$ is the mean value of the energy term of the models of the *i*^th^ domain, *n* is the number of the domains. Each energy term mean value for all domains was divided by the maximum mean absolute value of the energy term of all domains, so the *NMT* ranged from −1 to 1.

## Results

### Mirror structures

**Fig. 2a–c:**
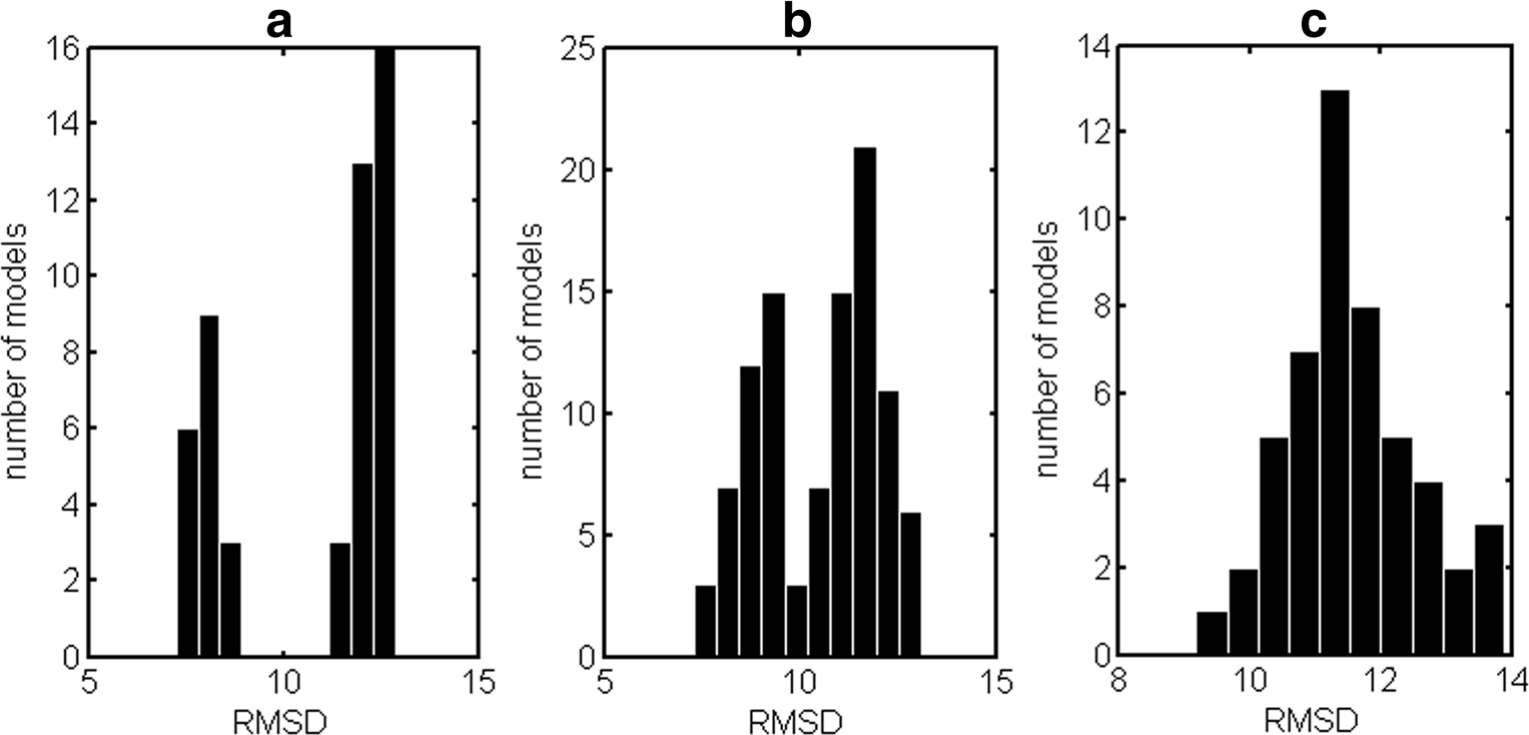
Root mean square deviation (RMSD) histograms demonstrating the possibility of distinguishing between properly oriented and mirror models. **a** Distinct (domain *d1ci4a*), **b** moderate (domain *d1a6qa1*), **c** indistinct (domain *d1fxkc*)

Protein structure reconstruction procedures based on contact maps provide either properly oriented models or mirror models. As a result, protein structural models can assume a wide range of RMSD values with respect to the target structure. Three general situations can be observed. Firstly, when high reconstruction accuracy is acquired then RMSD distribution allows for unambiguous separation of properly oriented and mirror models (Fig. 2a). Secondly, when moderate reconstruction accuracy is acquired, then properly oriented and mirror models overlap in the RMSD distribution, but they can be still separated (Fig. 2b). Finally, when reconstruction accuracy is low, then a single unimodal RMSD distribution is observed (Fig. 2c), which means that properly oriented and mirror structures are indistinguishable in terms of RMSD − all models are equally bad. Our aim was to characterize the differences between properly oriented and mirror models with regard to their energy terms.

**Fig. 3a–d:**
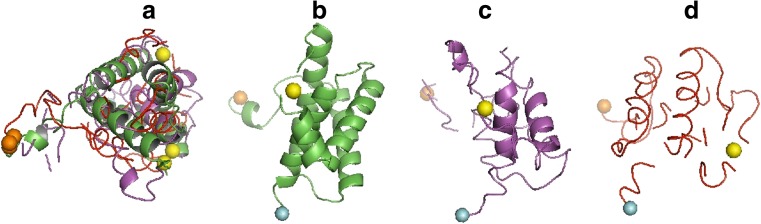
Structures of the domain *d1h99a1*. **a** Structure from SCOP (*green*), the properly oriented model (*magenta*) and the mirror model (*red*) oriented upon three points: the mass center, the N-terminus (*orange*) and the C-terminus (*blue*), where the yellow sphere is the C_α_ of ASN 63. **b** Structure from SCOP. **c** Properly oriented model. **d** Mirror model

To illustrate the problem of the mirror models, we chose the domain *d1h99a1*, for which we acquired a bimodal RMSD distribution. The domain *d1h99a1* is 115 residues long with four right-handed helices. Two exemplary models are shown in Fig. 3. Figure 3a shows the superposition of the properly oriented model, the mirror model, and the original SCOP structure. These structures are shown separately in Fig. 3b–d. Figure 3b shows the original SCOP structure. The model with the lower RMSD value (Fig. 3c) is properly oriented, with the right-handed helices such as in the SCOP structure. The helices of the model with high-value RMSD (Fig. 3d) are left-handed, and their relative arrangement resembles the mirror image of the SCOP structure.

**Fig. 4a–c:**
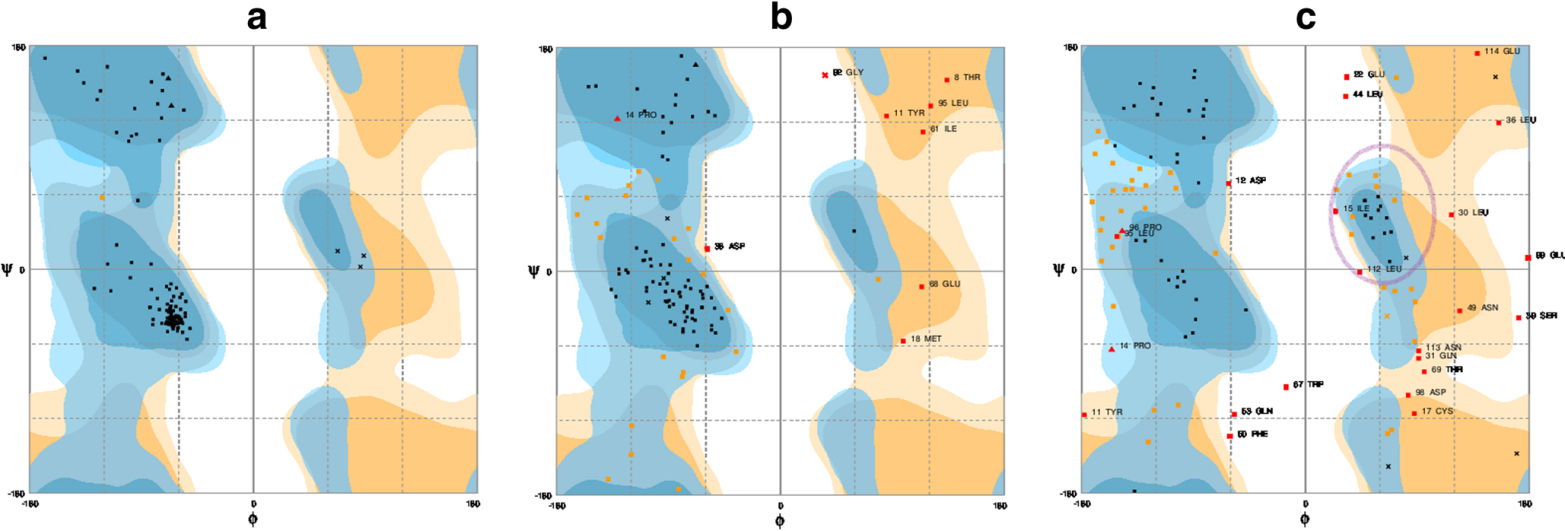
Ramachandran plots of the domain *d1h99a1*. **a** Structure from SCOP. **b** Properly oriented model. **c** Mirror model.* Blue* general favored region,* pale blue* allowed region,* orange* glycine favored region,* pale orange* glycine allowed region.* Black dots* residues in the favored region,* orange dots* residues in the allowed region,* red dots* residues in the outlier region.* Area circled in purple* left-handed alpha-helix region

To assess the number of residues of the domain *d1h99a1* that were arranged relative to each other inversely compared to the SCOP structure, we evaluated the Ramachandran plots of the models (Fig. 4, Table S1). The majority of the residues in the SCOP structure had negative dihedral angles, and were located in the right-handed alpha-helix region in the Ramachandran plot (Fig. 4a). In the original SCOP structure, 99.1 % of all residues were placed in the favored region and only 0.9 % in the allowed region. Moreover, only three residues had positive values of both dihedral angles, which positioned them in the left-handed alpha-helix region. In the model that was properly oriented, the proportion of residues located in the favored and allowed regions was slightly worse (Fig. 4b), i.e., 72.6 % in the favored region, and 19.25 % in the allowed region. A number of residues were distorted and fell into the outlier region. Nevertheless, most residues of the properly oriented model were located in the right-handed alpha-helix region without increasing the number of residues in the left-handed alpha-helix. In the mirror model, the residues had different locations in the Ramachandran plot (Fig. 4c). There were not as many residues in the right-handed alpha-helix region, while the number of residues in the left-handed alpha-helix region increased (purple circle in Fig. 4c). Despite the fact that the left-handed alpha-helix conformation could be also acceptable, the neighboring residues had to be rearranged in the modelling process to fit the unexpected structure. Therefore, only 46.4 % of residues were located in the favored region, while 36.3 % were in the allowed region, and 20.4 % fell into the outlier region. In summary, the proportion of residues that were located in the favored and allowed regions differed in the properly oriented model and the mirror model.

**Fig. 5a–c:**
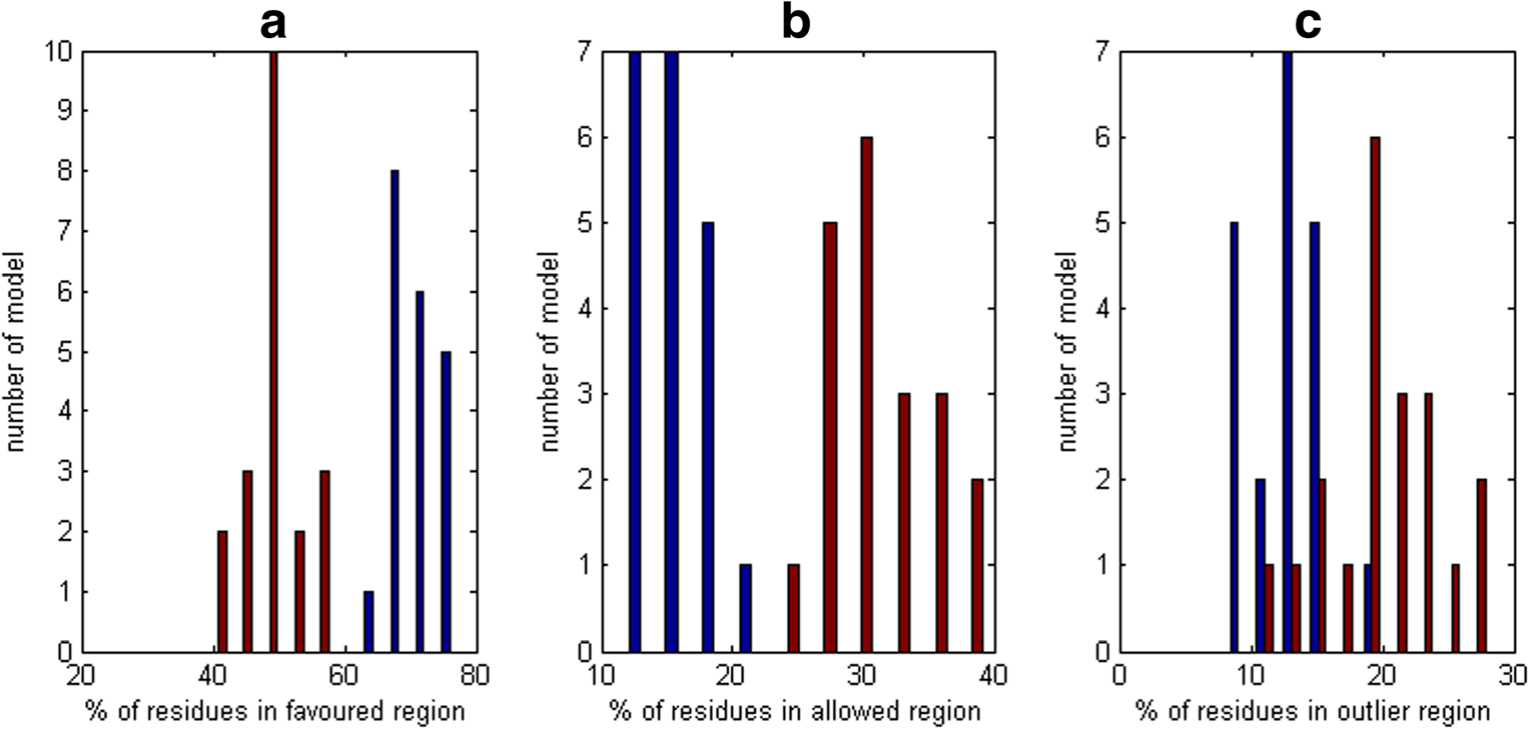
Differences in Ramachandran plots between properly oriented and mirror models of the domain *d1h99a1*. Histograms of percentage of residues in **a** favored region, **b** allowed region, and **c** outlier region.* Blue bars* properly oriented models,* red bars* mirror models

Others models of the domain *d1h99a1* confirmed this observation. The Ramachandran plots of the mirror models with greater RMSD values had many more residues in the left-handed alpha-helix region than the models with lower RMSDs. Also, the number of residues in the allowed region was greater for mirror models than for properly oriented models (Fig. 5a), while in the favored region this relationship was inversed (Fig. 5b).

### Energy terms

We showed that two non-overlapping distributions in the RMSD histograms resulted from differences in model orientation. An important question is if these mirror models are only bioinformatical artefacts or in fact represent competitive conformations of the target protein. Another issue is whether it is possible to separate these two types of model orientations without knowing the native structure; hence, the use of RMSD distributions. In order to address these questions, we analyzed the values of the energy terms of the models and compared them in the groups of properly oriented and mirror models.

**Fig. 6:**
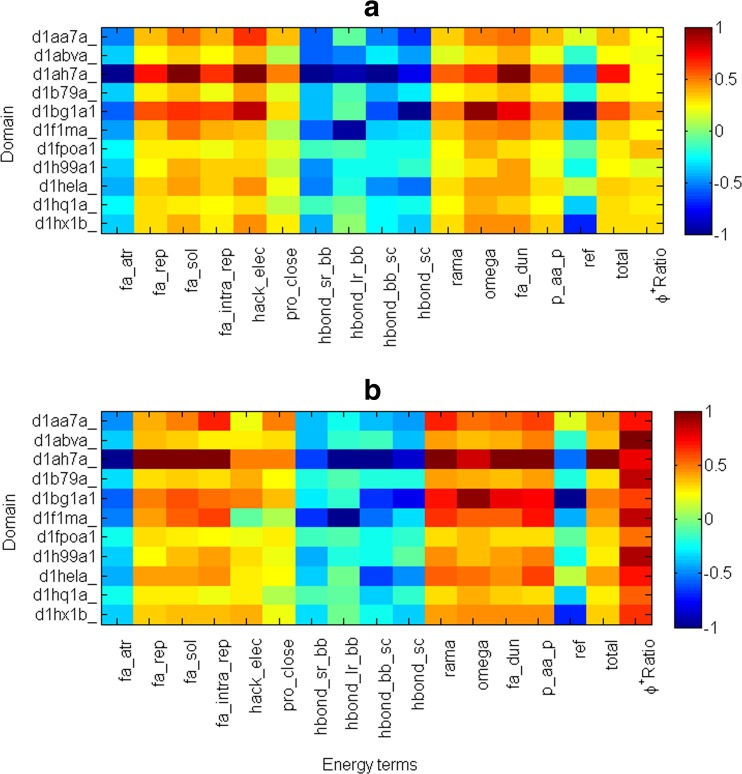
Map of the *NMT* (normalized energy term mean value, including *Φ* ^*+*^ *Ratio*) for **a** properly oriented models and **b** mirror models of 11 domains. Each square shows the *NMT* for models of each domain

We calculated the energy terms of all the models for 11 domains, then normalized the energy terms (for details see Materials and methods) and compared the values obtained in the groups of properly oriented and mirror models. The results revealed differences between the groups (Fig. 6).

Next, we examined which energy terms were significantly different for the properly oriented models and the mirror models for each domain. The results are shown in Table 2, where the symbol ‘•’ means that this energy term was significantly different for both types of model orientation. For each domain, we could indicate the energy term that was significantly different for the mirror models, but only two energy terms were significantly different for all domains: *rama* and *p_aa_p*. Both these energy terms correspond to dihedral angles in a model. *Rama* denotes Ramachandran preferences and *p_aa_p* determines the probability of the residue in the certain values of the dihedral angles. Also the calculated *Φ*^*+*^*Ratio* was significantly different for almost all domains, excluding one domain: *d1fpoa1*.

**Table 2:** Summary of the significant differences of the energy terms (including *Φ* ^*+*^ *Ratio*) between properly oriented models and their mirror images

Energy term domain	*fa_atr*	*fa_rep*	*fa_sol*	*fa_intra_rep*	*hack_elec*	*pro_close*	*hbond_sr_bb*	*hbond_lr_bb*	*hbond_bb_sc*	*hbond_sc*	*rama*	*omega*	*fa_dun*	*p_aa_p*	*ref*	*total*	*Φ* ^*+*^ *Ratio*
*d1aa7a_*	•^a^	•	•	•	•	•	•	•	•		•	•	•	•		•	•
*d1abva_*		•	•		•	•	•	•	•		•	•		•		•	•
*d1ah7a_*	•	•		•	•		•				•	•		•		•	•
*d1b79a_*	•	•	•	•		•	•		•		•	•		•		•	•
*d1bg1a1*	•	•	•		•		•				•			•		•	•
*d1f1ma_*		•	•	•	•				•		•	•		•		•	•
*d1fpoa1*					•	•					•			•			
*d1h99a1*	•		•	•		•				•	•	•		•			•
*d1he1a_*		•		•	•		•	•			•	•	•	•		•	•
*d1hq1a_*	•	•	•								•			•		•	•
*d1hx1b_*	•		•			•	•				•			•			•

^a^The symbol ‘•’ means that this energy term for this domain was significantly different between properly oriented and mirror models

**Fig. 7:**
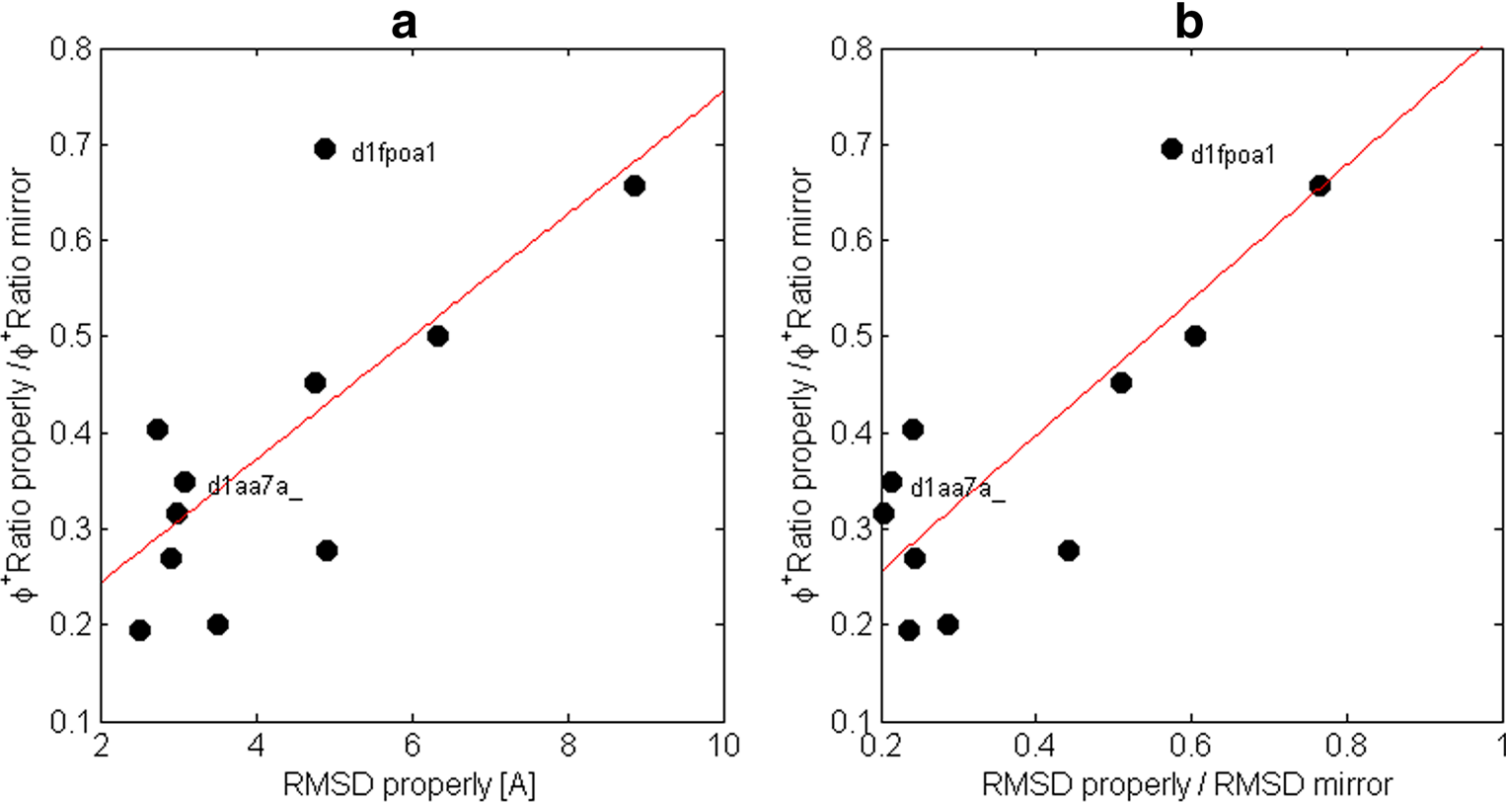
Comparison of the *Φ* ^*+*^ *Ratio* in properly oriented models and in mirror models for each domain to **a** the mean RMSDs of the properly oriented models for each domain, and **b** the ratios of the mean RMSDs of the properly oriented models and mirror models for each domain.* Red lines* trend lines

To explain the outlier result for the domain *d1fpoa1*, we first investigated if the number of residues could affect the proportion between the positive dihedral angles in the properly oriented and mirror models. No correlation was observed. Next, we evaluated if a degree of difficulty in modelling the structure, represented by the mean RMSD of the properly oriented models, correlated with the proportion between the positive values of the dihedral angles in the properly oriented and the mirror models (Fig. 7a). For domains with the right-handed alpha-helices, we observed that domains with lower RMSDs, i.e., the properly oriented models, had lower dihedral angles ratios. Therefore, there were more positive dihedral angles in the mirror models than in the properly oriented models. The Pearson correlation *r* of this relation was 0.76 with a* P*-value of 0.01. However, the mean RMSD of the properly oriented models of the domain *d1fpoa1* was 4.9 Å. It was located in the middle of the range of the mean RMSD of the other domains, which was from 2.5 Å to 8.9 Å. Nevertheless, the domain *d1fpoa1* was furthest away from the trend line. The lack of significant differences between the properly oriented and mirror models in terms of the dihedral angles did not ensue from the modelling difficulty. A strong correlation was also observed for the proportion between the positive values of the dihedral angles in the properly oriented models and in the mirror models, and the structural differences of the properly oriented and mirror models (Fig. 7b). The Pearson correlation *r* was 0.81 and the* P*-value less than 0.01, and the domain *d1fpoa1* was not located far from the trend line. The structural difference was defined as a ratio between the mean RMSD of the properly oriented models and the mean RMSD of the mirror models. This measure characterizes the possibility of differentiation between the models based on RMSD distribution. A ratio close to 1 suggests that the RMSDs of the models are equal in both groups. For these domains, the number of positive dihedral angles was also similar, regardless of the model orientation. Mean values of the number of positive dihedral angles of the domain *d1fpoa1* were 19 ± 5 in the properly oriented models and 21.5 ± 6 for the mirror models. Simultaneously, mean values of the *rama* energy term were 31.6 ± 4.8 and 37.4 ± 5.0 for properly oriented and mirror models, respectively. The mean values of the *p_aa_p* energy term were 23.3 ± 4.2 and 29.3 ± 4.7. The lack of significant differences in the number of the positive dihedral angles in both types of models of the domain *d1fpoa1* was caused by the high value of the standard deviation of the positive angles compared to the their mean values.

### Structural features of a domain and the number of the energy terms that were significantly different

**Fig. 8a–d:**
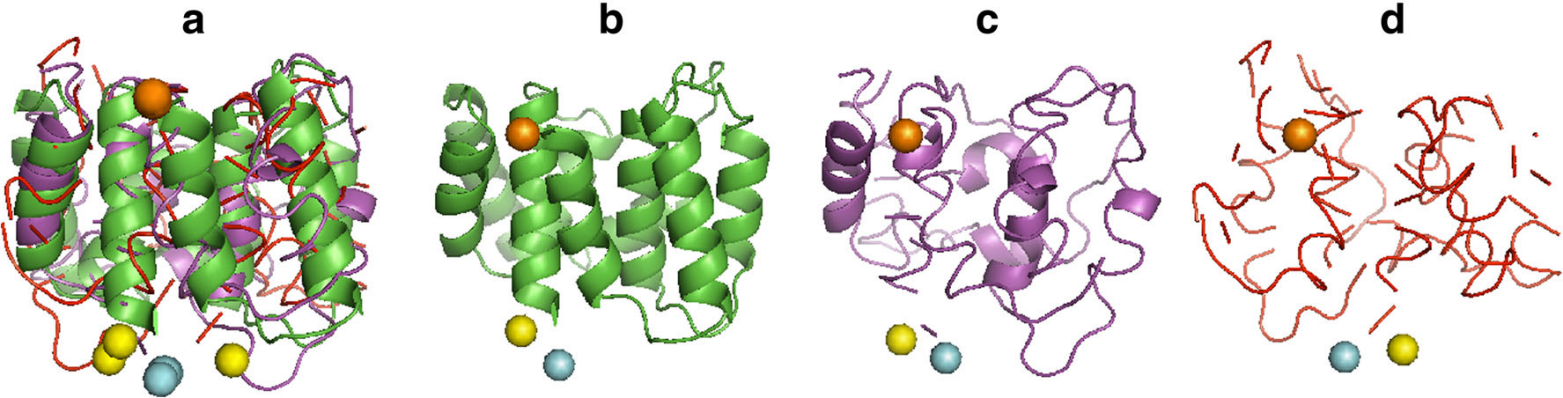
Structures of the domain *d1aa7a_*. **a** Structure from SCOP (*green*), the properly oriented model (*magenta*) and the mirror model (*red*) oriented upon three points: the mass center, N-terminus (*orange*) and C-terminus (*blue*), where the yellow sphere is the C_α_ of ASP 156. **b** Structure from SCOP. **c** Properly oriented model. **d** Mirror model

We also investigated the domain *d1aa7a_* (Fig. 8), which had the highest number of energy terms that were significantly different for the properly oriented and mirror models. The domain *d1aa7a_* is 158 residues long, and consists of two different subdomains. Each of those subdomains is built of four right-handed alpha-helices. The mean RMSD of the properly oriented models was 3.08 Å, and the ratio of the mean RMSD of the properly oriented models and the mirror models was 0.215 (Fig. 7). Only two energy terms could not be used for separating the mirror and properly oriented models: *hbond_sc* and *ref. Ref* energy term is the reference energy, which depends on the amino acid composition only, so it is the same in each model of the domain.

Moreover, the number of energy terms that were significantly different for properly oriented and mirror models was negatively correlated with the structural similarity between these two model groups, i.e. Pearson correlation *r* equalled −0.68 and the* P*-value was 0.02.

### Models with low reconstruction accuracy

In an additional study, we verified whether the features separating properly oriented and mirror models could also be applied for domains with overlapping RMSD profiles (Fig. 2b, c). The overall structural quality of those models was lower in comparison to the models in the main study, therefore the methodology for this study was changed slightly as follows: (1) model orientation was assessed not manually but based on superposition with the SCOP structure and with its ideal mirror image obtained by the symmetric reflection, (2) only 15 properly oriented and mirror models were required. We investigated 16 new domains. The energy term *rama* was significantly different for 11 out of 16 domains, and the energy term *p_aa_p* for 7 out of 16 domains. Lower structural quality of the models caused lower reliability of model orientation assessment. As a result, the statistical quality was decreased compared to the analysis of 11 domains in the main study. Therefore, for each domain, we additionally calculated the ratios of the mean values of the energy terms *rama* and *p_aa_p* between properly oriented and mirror models.

The results (data not shown) supported our conclusion regarding the usability of *rama* and *p_aa_p* energy terms because the ratios were lower than 1 for 14 out of 16 domains.

## Discussion

In this study we searched for a method of eliminating the mirror structures obtained in protein structure reconstruction from contact maps. Using methods that assess the structural features of models, such as the Ramachandran plot and the energy terms of the models, we investigated the differences between both types of model orientations. The Ramachandran plot of the models was a useful method to assess differences in structural orientation. The statistical analyses of the location of the residues in the various regions (favored, allowed and outlier) applied to the Ramachandran plot showed that the greatest differences occurred in the favored and allowed regions, rather than in the outlier region. This may be related to region size since the favored right-handed alpha-helix region is larger than the left-handed helix region. Hence, inaccuracies in the structure reconstruction are more acceptable in right-handed than in the left-handed alpha-helices.

Our *Φ*^*+*^*Ratio* for domains with right-handed alpha-helices was lower for properly oriented models and higher for their mirror images, because residues in right-handed alpha-helices have negative values of dihedral angles. In the opposite situation, when the native structure is rich in left-handed alpha-helices, the properly oriented models have a lot of positive dihedral angles and mirror models have more negative dihedral angles. In such cases, the *Φ*^*+*^*Ratio* still works but the value should be interpreted the opposite way: high ratio reflects a low RMSD value.

Even though the *Φ*^*+*^*Ratio* was sufficient to select mirror models for almost all studied domains, the energy terms that correspond with dihedral angles were standardized based on the structures from databases, and this may explain why they turned out to be a better indicator of the mirror orientation than only the number of positive dihedral angles.

The least separating energy term was *hbond_sc*, which is related to the hydrogen bond energy between side chains. This is a knowledge-based energy term, so its value depends on the statistical analysis of proteins in the databases. Hydrogen bonds between side chains stabilize right-handed and left-handed alpha-helixes, which may explain why we did not observe significant differences between properly oriented and mirror models. Moreover, every model, regardless of orientation (proper or mirror), whose structure was not crumpled and erroneously compacted, should have an appropriate value of *hbond_sc* energy term. Therefore, no significant differences of *hbond_sc* energy term values were observed for almost any domain, except domain *d1h99a1*.

## Conclusions

Our main goal was to detect structural properties and energy terms that could be useful in distinguishing between properly oriented and mirror models resulting from protein reconstruction based on contact maps. We analyzed structural models of a set of SCOP domains rich in right-handed helices. The models were acquired with a reconstruction protocol based on inter-residue contact maps. The set of models consisted of properly oriented and mirror structures. The main group of models was assessed visually with regard to their proper or mirror orientation. The properly oriented models had lower RMSD than the mirror models, but the quality of both types of models could be similar. The information about the dihedral angles of residues in the models could be useful in distinguishing between the two types of orientation. However, we showed that the *Φ*^*+*^*Ratio* is not sufficient for all domains, and it may depend on the modelling difficulty of the structure.

Our idea was to use the information about dihedral angles regarding statistical preferences of the proteins gathered in databases. For this reason, energy terms such as *p_aa_p* and *rama* could be used because they are based on dihedral angles. Significant differences in the energy term values of properly oriented and mirror models were also observed for other energy terms, but not for all domains. Despite the fact that both types of models are structurally different from each other, they could have similar total energies. Our study found three domains that did not show significant differences in total energy values for properly oriented and mirror models. We also showed a negative correlation between the number of energy terms that were significantly different for properly oriented and mirror models and the structural similarity of two types of model orientations. In other words, it is easier to distinguish proper and mirror models if the reconstruction procedure provides us with high quality structures. Moreover, we note that other research showed that mirror models may provide competitive forms of the native protein, rather than representing only bioinformatical artefacts [16, 17].

## Electronic supplementary material

Below is the link to the electronic supplementary material.

Table S1 (DOCX 14 kb)
