# Supplementary material for: Applying PyRosetta molecular energies to separate properly oriented protein models from mirror models, obtained from contact maps
Source: J Mol Model. 2016 Apr 23;22:111. doi: 10.1007/s00894-016-2975-3 (PMC4842210; doi:10.1007/s00894-016-2975-3)
Supplement: Supplementary file 1 — (DOCX 14 kb) [file 894_2016_2975_MOESM1_ESM.docx]

Table S1 The summary of the Ramachandran analysis for *d1h99a1* domain.

| No model | %Number of residues in | | |
| --- | --- | --- | --- |
|  | favoured region | allowed region | outlier region |
| Properly oriented models | | | |
| 1 | 68.1 | 18.6 | 13.3 |
| 2 | 69.0 | 15.9 | 15.0 |
| 3 | 66.4 | 17.7 | 15.9 |
| 4 | 74.3 | 14.2 | 11.5 |
| 5 | 73.5 | 12.4 | 14.2 |
| 6 | 72.6 | 14.2 | 13.3 |
| 7 | 74.3 | 16.8 | 8.8 |
| 8 | 69.9 | 15.0 | 15.0 |
| 9 | 77.0 | 15.0 | 8.0 |
| 10 | 64.6 | 22.1 | 13.3 |
| 11 | 69.9 | 18.6 | 11.5 |
| 12 | 69.0 | 15.0 | 15.9 |
| 13 | 69.9 | 17.7 | 12.4 |
| 14 | 70.8 | 16.8 | 12.4 |
| 15 | 69.0 | 11.5 | 19.5 |
| 16 | 74.3 | 16.8 | 8.8 |
| 17 | 73.5 | 13.3 | 13.3 |
| 18 | 73.5 | 13.3 | 13.3 |
| 19 | 77.9 | 13.3 | 8.8 |
| 20 | 72.6 | 19.5 | 8.0 |
| **mean** | **71.5 ± 3.4** | **15.9 ± 2.7** | **12.6 ± 3.0** |
| Mirror models | | | |
| 21 | 47.8 | 33.6 | 18.6 |
| 22 | 53.1 | 25.7 | 21.2 |
| 23 | 47.8 | 28.3 | 23.9 |
| 24 | 48.7 | 23.0 | 28.3 |
| 25 | 43.4 | 36.3 | 20.4 |
| 26 | 49.6 | 30.1 | 20.4 |
| 27 | 47.8 | 29.2 | 23.0 |
| 28 | 47.8 | 29.2 | 23.0 |
| 29 | 57.5 | 28.3 | 14.2 |
| 30 | 52.2 | 28.3 | 19.5 |
| 31 | 56.6 | 28.3 | 15.0 |
| 32 | 38.9 | 34.5 | 26.5 |
| 33 | 47.8 | 38.9 | 13.3 |
| 34 | 41.6 | 39.8 | 18.6 |
| 35 | 54.9 | 33.6 | 11.5 |
| 36 | 44.2 | 30.1 | 25.7 |
| 37 | 46.0 | 35.4 | 18.6 |
| 38 | 50.4 | 30.1 | 19.5 |
| 39 | 50.4 | 30.1 | 19.5 |
| 40 | 49.6 | 32.7 | 17.7 |
| **mean** | **48.8 ± 4.7** | **31.3 ± 4.3** | **19.9 ± 4.4** |
